# The Prognostic and Discriminatory Utility of the Clinical Frailty Scale and Modified Frailty Index Compared to Age

**DOI:** 10.3390/geriatrics7050087

**Published:** 2022-08-24

**Authors:** Ben Carter, Victoria L. Keevil, Atul Anand, Christopher N. Osuafor, Robert J. B. Goudie, Jacobus Preller, Matthew Lowry, Sarah Clunie, Susan D. Shenkin, Kathryn McCarthy, Jonathan Hewitt, Terence J. Quinn

**Affiliations:** 1 Department of Biostatistics and Health Informatics, Institute of Psychiatry, Psychology and Neuroscience, King’s College London, London SE5 8AF, UK; 2 Department of Medicine for the Elderly, Addenbrooke’s Hospital, Cambridge University Hospitals NHS Foundation Trust, Hills Road, Cambridge CB2 0QQ, UK; 3 Department of Medicine, University of Cambridge, Cambridge CB2 0QQ, UK; 4 Centre for Cardiovascular Science, Queens Medical Research Institute, University of Edinburgh, Edinburgh EH16 4TJ, UK; 5 Department of Clinical Neurosciences, University of Cambridge, Cambridge CB2 0QQ, UK; 6 Medical Research Council Biostatistics Unit, University of Cambridge, Cambridge CB2 0SR, UK; 7 Department of Acute Internal Medicine and Intensive Care, Cambridge University Hospitals NHS Foundation Trust, Cambridge CB2 0QQ, UK; 8 Geriatric Medicine, Usher Institute, University of Edinburgh; Edinburgh EH16 4UX, UK; 9 Department of Surgery and Care of the Elderly, Southmead Hospital, North Bristol NHS Trust, Bristol BS10 5NB, UK; 10 Division of Population Medicine, Cardiff University, Cardiff CF10 3AT, UK; 11 Institute of Cardiovascular and Medical Sciences, University of Glasgow, Glasgow G12 8TA, UK

**Keywords:** older people, COVID-19, frailty, clinical frailty scale, modified frailty index, prognosis

## Abstract

**Background**: There is no consensus on the optimal method for the assessment of frailty. We compared the prognostic utility of two approaches (modified Frailty Index [mFI], Clinical Frailty Scale [CFS]) in older adults (≥65 years) hospitalised with COVID-19 versus age. **Methods:** We used a test and validation cohort that enrolled participants hospitalised with COVID-19 between 27 February and 30 June 2020. Multivariable mixed-effects logistic modelling was undertaken, with 28-day mortality as the primary outcome. Nested models were compared between a base model, age and frailty assessments using likelihood ratio testing (LRT) and an area under the receiver operating curves (AUROC). **Results:** The primary cohort enrolled 998 participants from 13 centres. The median age was 80 (range:65–101), 453 (45%) were female, and 377 (37.8%) died within 28 days. The sample was replicated in a validation cohort of two additional centres (n = 672) with similar characteristics. In the primary cohort, both mFI and CFS were associated with mortality in the base models. There was improved precision when fitting CFS to the base model +mFI (LRT = 25.87, *p* < 0.001); however, there was no improvement when fitting mFI to the base model +CFS (LRT = 1.99, *p* = 0.16). AUROC suggested increased discrimination when fitting CFS compared to age (*p* = 0.02) and age +mFI (*p* = 0.03). In contrast, the mFI offered no improved discrimination in any comparison (*p* > 0.05). Similar findings were seen in the validation cohort. **Conclusions:** These observations suggest the CFS has superior prognostic value to mFI in predicting mortality following COVID-19. Our data do not support the use of the mFI as a tool to aid clinical decision-making and prognosis.

## 1. Introduction

Frailty is a state of increased vulnerability to external stressors such as illness [1]. There is growing recognition of the prevalence and importance of frailty in healthcare. Differing methods for the identification and quantification of frailty have evolved, and at present, there is no consensus on the optimal approach. The most commonly described approaches within frailty research are a frailty index (FI), which is a quantification of the cumulative burden of health deficits [2], or a phenotypic approach based on traits such as weakness, slowness, and low physical activity [3]. The first papers describing the FI approach used 70 different items in the index [4], limiting direct application to clinical care. Similarly, measures such as grip strength and gait speed required to derive the frailty phenotype need additional equipment and clinical assessment.

Given the constraints of these research tools, there has been growing interest in pragmatic and brief clinical assessments of frailty. Refinements to the FI have seen preserved validity with fewer items [5]. A modified FI (mFI) that uses only five items frequently collected in routine health datasets has been validated, although predominantly in surgical settings [6,7]. There are certain reasons to prefer a FI approach for research and policy. The FI metric can be retrospectively derived from routinely available clinical data and is an objective measure, but it may be biased towards over-estimating an individual’s frailty [8].

The Clinical Frailty Scale (CFS) is based upon direct assessment and requires clinical judgement and is performed in-person, based on the person’s physical status two weeks prior, but can also be assessed by a trained clinician using a case-note review [9]. More recently the recording of CFS has widened [10].

The COVID-19 pandemic has shown that people living with frailty have poor outcomes following COVID-19 infection [11,12,13]. In response, guidance suggested that the assessment of frailty should be used to inform resource allocation decisions. In the UK, the National Institute of Clinical Excellence went further still, offering a recommendation that frailty be assessed using the CFS to guide clinical decision-making. This highlighted many fundamental questions around clinical frailty scoring, such as the mFI and CFS that add more information about age.

In practice, the identification of frailty alongside age is often used to support healthcare decisions as part of a holistic patient assessment [14]. Thus, quantifying the prognostic utility of differing frailty tools would allow for the comparison and could assist in choosing the best measure.

### Objectives

The aim of this study was to compare the prognostic utility of mFI, CFS, and age. Specifically, we compare mFI and CFS against age for mortality and length of stay and compare the discrimination between each.

## 2. Methods

### 2.1. Study Design

Our primary dataset was a prospective cohort study [11]. We created a validation cohort from two studies based in Cambridge [12] and Edinburgh. All studies were designed with the primary aim to assess the prognostic utility of frailty to predict mortality for patients hospitalised with COVID-19 and included patients within the first wave of the pandemic. We followed Strengthening the Reporting of Observational Studies in Epidemiology (STROBE guidance) for reporting [15].

### 2.2. Ethics and Data Availability

Authority in the UK to conduct the primary cohort was granted by the Health Research Authority (20/HRA/1898). The validation cohort study based in Cambridge was approved by the Institutional Review Board of West Midlands-Coventry and Warwickshire Research Ethics Committee (REC number 20/WM/0125, Protocol 1.1 Amendment 1, 24 April 2020). The validation cohort study in Edinburgh was reviewed by the DataLoch ethics review panel and approved under generic REC permissions granted to the Lothian Research Safe Haven (17/NS/0072).

### 2.3. Population

Our primary cohort was a prospective cohort that included sequential hospital inpatients (The COPE Study), from 27 February to 30 June 2020 with COVID-19 from 13 hospital sites across the UK and Italy [11,16]. Only participants ≥ 65 years old were included.

The validation cohorts were prospectively collected using secondary care data from two UK University Hospital sites. The first validation cohort included patients admitted to three acute hospital sites in Edinburgh, with routine clinical and additional manually collected data extracted via the NHS Lothian DataLoch facility (University of Edinburgh). The second dataset came from routine data recorded in an electronic health record at Cambridge University Hospital’s NHS Foundation Trust [12]. Both included consecutive hospital in-patients with COVID-19 aged ≥ 65 years (Cambridge: 1 March 2020 to 15 May 2020; Edinburgh and NHS Lothian:1 March 2020 to 30 June 2020). The two cohorts were combined for the validation analysis.

### 2.4. Prognostic Factors

**Modified Frailty Index (mFI):** The modified frailty index is based on the cumulative deficit model where the more comorbidities present, the higher the frailty index (FI). For this work, we used the mFI [6] since it has been specifically designed for the retrospective assessment using clinical datasets and electronic health records, and is validated in various settings [7,17]. The mFI includes chronic heart failure, chronic obstructive pulmonary disease, diabetes mellitus, being on treatment for hypertension, and functional dependence as the component deficits. This gives an mFI range of 0–1, with each contributing domain assigned a score of 0.2. For analyses, mFI was categorised as not frail (mFI < 0.4) and frail (mFI ≥ 0.4).

**Clinical Frailty Scale:** The Clinical Frailty Scale (CFS) takes information from an unstructured clinical encounter and is assessed using an ordinal hierarchical scale [17]. Patients determined as terminally ill (CFS 9) were excluded from this analysis. In keeping with other studies using CFS as a prognostic tool, for analyses, the CFS score was categorised as not frail (CFS 1–4) and frail (CFS 5–8) within the primary analysis and divided into four groups at CFS scores of 1–4, 5, 6, and 7–8 for the secondary analyses.

In the primary dataset, all CFS data were collected prospectively, through in-person assessment by trained clinicians at participating centres. In the Edinburgh and Cambridge datasets, the CFS was recorded during patient admission, but where this was not possible the scale was extracted directly from notes using clinical judgement [18].

**Age:** Age was categorised into ten-year bands from 65 to 94 years old. Patients aged 95 or older were grouped together.

### 2.5. Outcomes

Our primary outcome was mortality at Day 28, measured from admission to hospital, or from the date of positive COVID-19 diagnosis for those patients with a length of stay greater than 5 days pre-diagnosis (i.e., presumed nosocomial infection). Patients discharged prior to Day 28 were imputed as survivors at the endpoint.

Our secondary outcome was prolonged admission status, defined as the length of stay longer than 14 days from the date of COVID-19 diagnosis (or inpatient mortality prior to this).

### 2.6. Statistical Analyses

We limited analyses to patients aged 65 years or over, who were not terminally ill (CFS 9). In the primary cohort, correlations were fitted to compare mFI, CFS, and age category. Age group, CFS, and mFI were compared using pairwise correlations and Altman–Bland plots.

A mixed-effects multivariable logistic regression was fitted to Day-28 mortality, where each hospital site was fitted with a random intercept to account for hospital level variability. Fixed effects included pre-specified covariates agreed by the investigators to be associated with COVID-19 outcomes in a base model: sex, CRP (elevated ≥ 40 mg/mL [19]), and smoking status. CFS was assessed as both dichotomous and ordinal data. Nested models were fitted independently for mFI, CFS, and age, with comparison using likelihood ratio testing [LRT].

To assess discrimination, we used the C-statistic, the area under the receiver-operating characteristic curve (AUROC) metrics to assess the discriminative ability of each model (mFI, CFS, age) adjusted for sex, CRP, and smoking. We compared AUROC values using the non-parametric method described by DeLong [20] and then in 2000 bootstrapped samples. Equivalent analyses were reported for the secondary outcome of prolonged admission status.

Discrimination and calibration assessments were assessed for the validation cohort by applying the model coefficients obtained from the primary dataset at an individual patient level. To assess calibration, we divided the populations into equally sized groups and visually compared predicted against observed risk. As a further test, we used the Hosmer Lemeshow test where a *p* < 0.05 with a high χ^2^ statistic was taken as evidence of poor calibration.

All analyses were conducted using Stata software (version 16) and R (version 3.6.3, London).

## 3. Results

We included 998 patients from the COPE cohort. Patient characteristics can be found in Table 1. The median age was 80 (range:65–101), and 45.4% (n = 453) were female. In-patient mortality was 39.1% (n = 390) with 37.8% (n = 377) dead by day 28. Prolonged admission (beyond day 14) was recorded in 73.2% (n = 731). Using the binary mFI threshold at ≥ 0.4, 48.7% (n = 486) were classified as frail. Using the binary CFS threshold at ≥ 5, 63.1% (n = 630) patients were considered frail. There was moderate correlation between CFS and mFI (r = 0.27) and CFS and age (r = 0.38). There was a weak correlation between mFI and age (0.12) (Supplementary Tables S1–S3, Supplementary Figure S1).

### 3.1. Day 28 Mortality

The mFI (binary) was associated with mortality, compared to mFI 0–0.2, mFI ≥ 0.4 aOR = 1.53 (95%CI: 1.16–2.03, Table 2). CFS (binary) was associated with mortality, compared to CFS 1–4, CFS 5–8 aOR = 2.12 (95%CI: 1.56–2.87).

Age was associated with mortality in our logistic regression models. Compared to 65–74 years, older age groups were more likely to die: ages 75–84 aOR = 2.01 (95%CI: 1.43–2.84); 85 to 94 aOR = 2.58 (95%CI: 1.75–3.81), and 95 or older aOR = 5.49 (95%CI: 2.09–14.39; Table 2).

In the secondary analysis fitting CFS as hierarchical categories, 1–4 (not frail), 5 (mildly frail), 6 (moderately frail), and 7–8 (severely frail), CFS was associated with mortality showing a ‘dose response’ relationship, compared to CFS 1–4: CFS 5 aOR = 1.42 (95%CI: 0.93–2.16); CFS 6 aOR = 2.24 (95%CI: 1.52–3.30); CFS 7–8 aOR = 2.78 (95%CI: 1.90–4.07). Comparing nested models, there was evidence for improved performance between the base model with: age (LRTχ^2^ = 31.96; *p* < 0.001); mFI (LRTχ^2^ = 9.11, *p* = 0.003), and CFS (LRTχ^2^ = 32.99, *p* < 0.001). Comparing the addition of CFS and mFI to the base model + age there was further improvement in precision to the model fit for CFS (LRTχ^2^ = 21.4, *p* < 0.001) and mFI (LRTχ^2^ = 6.11, *p* = 0.01). There was also an improvement of precision fitting CFS in addition to base model + mFI (LRTχ^2^ = 25.87, *p* < 0.001); however, there was no improvement with fitting mFI after base model +CFS (LRTχ^2^ = 1.99, *p* = 0.16).

### 3.2. Prolonged Admission

The mFI (binary) was associated with a longer length of stay, compared to mFI 0–0.2, mFI ≥ 0.4 aOR = 1.39 (95%CI: 1.04–1.88). CFS (binary) was associated with a longer length of stay, compared to CFS 1–4, CFS 5–8 aOR = 2.44 (95%CI: 1.79–3.30). There was an inconsistent association between age and increased length of stay, with substantial imprecision in estimates. Compared to 65–74 years: ages 75–84 aOR = 1.23 (95%CI: 0.88–1.71); 85 to 94 aOR = 2.49 (95%CI: 1.64–3.79), and 95 or older aOR = 2.19 (95%CI: 0.70–6.84) (Table 3).

In the secondary analysis fitting CFS as hierarchical categories, higher CFS scores were also associated with longer length of stay, compared to CFS 1–4: CFS 5 aOR = 1.26 (95%CI: 0.93–2.16); CFS 6 aOR = 3.14 (95%CI: 2.04–4.85); CFS 7–8 aOR = 3.75 (95%CI: 2.41–5.83). By comparing nested models, there was evidence of an improved performance between the base model with age (LRTχ^2^ = 21.18; *p* < 0.001), CFS (LRTχ^2^ = 52.80, *p* < 0.001), and mFI (LRTχ^2^ = 4.84, *p* = 0.03). Comparing the base model + age group compared to the addition of CFS, there was a very strong improvement in precision with CFS (LRTχ^2^ = 39.91, *p* < 0.001), but no improvement was found when mFI was included with the base model + age (LRTχ^2^ = 2.87, *p* = 0.09). In the comparison of CFS and mFI directly, there was an improvement in precision fitting with the addition of CFS after mFI (LRTχ^2^ = 48.00, *p* < 0.001), but no improvement of fitting mFI after CFS (LRTχ^2^ = 0.84, *p* = 0.84).

### 3.3. Discrimination

**Figure 1:**
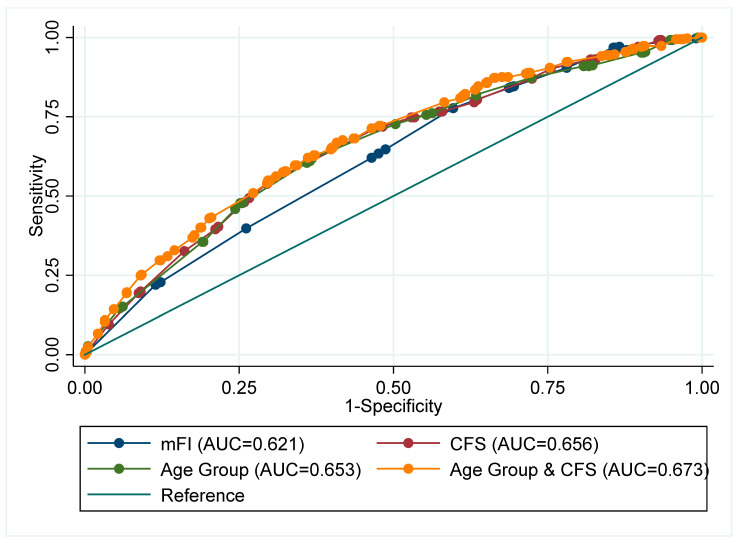
Receiver operating Area Under the Curve (AUC) for Day-28 mortality comparison of adjusted analyses fitting: Sex, smoking status, and elevated CRP.

For 28-Day mortality, the AUROC was 0.62 (95%CI 0.59–0.66) for mFI, 0.66 (95%CI 0.62–0.69) for CFS, and 0.65 (95%CI 0.62–0.69) for age (Supplementary Table S4 and Figure 1). Comparing the AUROC, the CFS consistently offered greater discrimination compared to both age (*p* = 0.02), and age + mFI (*p* = 0.03). Whereas the mFI offered no improved discrimination in any comparison (*p* > 0.05).

For prolonged admission, the AUROC was 0.56 (95%CI 0.52–0.60) for mFI, 0.66 (95%CI 0.62–0.70) for CFS, and 0.60 (95%CI: 0.56–0.63) for age (Supplementary Table S5). Comparing the AUROC, the CFS offered improved discrimination compared to age (*p* < 0.001) and age + mFI (*p* < 0.001), but mFI was not found to offer improved discrimination after accounting for CFS (*p* = 0.055).

### 3.4. Validation Cohort

We included 672 patients from the validation cohort (Edinburgh 461; Cambridge 211). Patient characteristics can be found in Supplementary Table S5 and were broadly similar to the primary cohort. The primary outcome of day 28 mortality occurred in 249 (37%) patients. The prolonged admission endpoint occurred in 444 (66%) patients.

Using the binary mFI threshold at ≥ 0.4, 338 (50%) were classified as frail. Using the binary CFS threshold at ≥ 5, 417 (62%) patients were considered frail. When the COPE-derived model including age as a predictor of mortality was applied to the validation cohort, AUROC for the mFI model was 0.59 (95%CI: 0.55–0.64); for the CFS model, the AUROC was 0.64 (95%CI: 0.60–0.68); and for age, the AUROC was 0.63 (95%CI: 0.59–0.67). Comparing the AUROC values there were significant differences in favour of the model including CFS compared to mFI (*p* = 0.005). However, the CFS model did not improve on the discrimination provided by age adjustment (*p* = 0.57). The mFI did not demonstrate any improvement beyond the base model including sex, smoking status, and elevated CRP (*p* = 0.91). Thus, the CFS offered improved utility compared to the mFI.

For the secondary endpoint of prolonged admission, there were similar findings and once more the mFI did not add to the base model discrimination (*p* = 0.12). Visual inspection suggested adequate calibration for all three models for both outcomes (Supplementary Figures S2 and S3). The Hosmer–Lemeshow Goodness of Fit testing results suggested adequate calibration of all models (Supplementary Table S6).

## 4. Discussion

The study, including 1672 inpatients hospitalised with COVID-19, confirms that frailty is strongly associated with adverse outcomes. Frailty assessed using the CFS exhibited an improved model precision and discrimination compared to frailty assessment using the mFI. This was true for both 28-day mortality and prolonged length of stay. The CFS offered improved prognostic utility to both age and mFI for mortality and prolonged length of stay, whereas the mFI did not. Based on these data, CFS seems the preferred approach to frailty assessment in this patient population.

Our finding of differential prognostic utility between the ‘subjective’ CFS based on clinical judgement and the ‘objective’ mFI based only on information in health records, would support the policy instituted in the UK and other countries of routine measurement of CFS in unscheduled older adult admissions [21,22]. Large-scale, robust studies have reported significant associations between frailty and outcomes [11,12,13]. The studies reporting the strongest associations have tended to be based on prospectively collected CFS assessments. In our datasets, patterns of association were similar for both the primary (prospective in-person evaluation of CFS) and validation (mix of in-person and case note CFS) cohorts. These results suggest that the CFS derived through routine clinical care, either scored by the treating team or through case-note review, offers similar prognostic utility to an in-person CFS assessment performed as part of a prospective research study. We suggest that the CFS should be derived using all available clinical data, but in-person assessment by the scorer is not mandatory for a valid assessment.

The differential prognostic utility between the clinical frailty assessment and the frailty index may have other explanations. The frailty index that we used, although validated and used in practice, has fewer elements that contribute to scoring than the classical indices. It is possible that CFS and mFI are measuring differing constructs. We note the modest agreement between the two metrics in our dataset, where we found, at best, a weak correlation between all measures, and both chronological age and mFI appeared to bias individuals at a higher category of frailty. These findings align with other studies that have suggested that different approaches to frailty scoring are not always directly comparable [8,23].

While the associations of frailty measures and outcomes were robust, the prognostic utility of the tools was far from perfect, and we would not support a reductionist approach of relying on frailty assessments alone to inform complex decision-making. The simple construct of chronological age also had reasonable prognostic utility and these results are a reminder of the prognostic importance of age. While our focus was the prognosis, assessing frailty has utility beyond simple early prognostication. An awareness of frailty and incorporation into care pathways is recognised as best practice and should be encouraged. It is a clinical indicator that can be used to help healthcare professionals anticipate patients’ needs and proactively consider advanced care planning in discussion with the patient and their priority for specialist hospital services which can improve outcomes in older patients [24]. Other studies have found the impact of having a CFS assessment led to a reduction in mortality [25].

Our study had inherent strengths and limitations. We had access to a large and well-phenotyped cohort of older adults and were able to validate our findings in an independent population. The nature of our primary and validation data allowed us to assess the novel and important question of the approach to CFS derivation. Although the sample sizes used were large, the sample sizes required for prognostic research are substantial and any modest differences found in the validation cohorts were likely due to the uncertainty of the estimates and power. Further weaknesses included that we were not able to differentiate between mortality due to COVID-19 as the primary cause or measure other patient-level covariates such as the Charlson Comorbidity Index, or the number of medications. Our study was designed to assess the comparative utility of frailty as assessed using different methods [26]. In practice, clinicians are more likely to combine prognosis assessments with other clinical and demographic factors to inform a holistic assessment of potential outcomes.

These data have implications for practice and future research. Where possible, we would support the clinical assessment of frailty with a tool such as CFS and we also encourage the collection of these frailty data into electronic resources for research and service improvement. We also recognise that mortality and length of stay are blunt measures of outcome and future research may wish to consider outcomes that are important to older adults and can be derived at scale, such as return home, disability, dementia, and institutionalisation [27,28,29].

## 5. Conclusions

Frailty was associated with poor outcomes following COVID-19, and the CFS was superior to the mFI. Frailty assessment benefits from clinical interpretation. Although direct, in-person assessments may not always be required. However, CFS alone is not sufficient to make decisions on treatment, and other factors need to be considered.

## Figures and Tables

**Table 1:** Characteristics of the included patients in the primary cohort.

	Day 28 Mortality		
	Dead	Alive	Total
Sites	n = 377 (37.8%)	n = 621 (62.2%)	n = 998 (%)
Age			
65–69 yrs	23 (19.8)	93 (80.2)	116 (11.6)
70–74 yrs	58 (33.9)	113 (66.1)	171 (17.1)
75–79 yrs	81 (39.1)	126 (58.3)	207 (20.7)
80–84 yrs	90 (41.7)	92 (59.7)	216 (21.6)
85–89 yrs	62 (40.3)	92 (59.7	154 (15.4)
90 yrs or older	63 (47.0)	71 (53.0)	134 (13.4)
Female	152 (33.6)	301 (66.5)	453 (45.4)
Current smokers	17 (32.1)	36 (67.8)	53 (5.3)
Diabetes	123 (38.9)	193 (61.8)	316 (31.7)
Hypertension	55 (38.7)	87 (61.3)	142 (14.2)
Hypertension (on treatment)	157 (37.0)	268 (63.1)	425 (42.6)
Coronary Artery Disease	123 (44.7)	152 (55.3)	275 (27.6)
Elevated CRP (>40)	294 (44.1)	372 (55.9)	666 (66.7)
eGFR ≥ 60	167 (31.8)	359 (68.3)	526 (52.7)
COPD	90 (45.2)	109 (54.8)	199 (19.9)
Heart Failure	84 (49.7)	85 (50.3)	169 (16.9)
Modified Frailty Index Items (mFI)			
0	95 (37.0)	162 (63.0)	257 (25.8)
1	80 (31.4)	175 (68.6)	255 (25.6)
2	119 (37.3)	200 (62.7)	319 (32.0)
3	68 (50.4)	67 (49.6)	135 (13.5)
4	10 (40.0)	15 (60.0)	25 (2.5)
5	5 (71.4)	2 (28.6)	7 (0.7)
Clinical Frailty Scale (CFS)			
1, Very Fit	6 (30.0)	14 (70.0)	20 (2.0)
2, Fit	18 (29.5)	43 (70.5)	61 (6.11)
3, Managing well	39 (27.1)	105 (72.9)	144 (14.4)
4, Very Mildly frail	50 (35.0)	93 (65.0)	143 (14.3)
5, Mildly frail	56 (34.8)	105 (65.2)	161 (16.3)
6, Moderately Frail	89 (40.8)	129 (59.2)	218 (21.8)
7, Severely frail	87 (44.4)	109 (55.6)	196 (19.6)
8, Very severely frail	32 (58.2)	23 (41.8)	55 (5.5)

**Table 2:** Day-28 mortality, mixed effects logistic regression, presenting the Odds ratio (OR) and adjusted OR (aOR) adjusted for the Clinical Frailty Scale (CFS), modified Frailty Index (mFI), and age group.

	Crude Odds Ratio (OR)		Base Model Adjusted OR (aOR) ^&^		Base + Age aOR		Base + CFS (Binary) aOR		Base + mFI (Binary) aOR	
	OR (95%CI)	*p*	aOR (95%CI)	*p*	aOR (95%CI)	*p*	aOR (95%CI)	*p*	aOR (95%CI)	*p*
Constant	0.52 (0.36–0.75)	<0.001	0.25 (0.16–0.40)	<0.001	0.126 (0.07–0.22)	<0.001	0.145 (0.09–0.24)	<0.001	0.199 (0.12–0.32)	<0.001
Sex (Female)	Ref	-								
Male	1.32 (1.01–1.72)	0.044	1.26 (0.96–1.65)	0.10	1.33 (1.01–1.76)	0.043	1.41 (1.07–1.87)	0.016	1.30 (0.99–1.72)	0.06
Smoking (Never/Ex)	Ref	-								
Current smoker	0.78 (0.42–1.45)	0.44	0.77 (0.41–1.44)	0.41	0.81 (0.43–1.54)	0.52	0.69 (0.36–1.29)	0.25	0.76 (0.41–1.43)	0.40
Elevated CRP (≥40)	2.37 (1.75–3.21)	<0.001	2.33 (1.72–3.16)	<0.001	2.56 (1.88–3.49)	<0.001	2.47 (1.81–3.36)	<0.001	2.34 (1.73–3.18)	<0.001
Age Group (65–74)										
75–84 yrs	1.87 (1.34–2.62)	<0.001			2.01 (1.43–2.84)	<0.001				
85–94 yrs	2.18 (1.50–3.17)	<0.001			2.58 (1.75–3.81)	<0.001				
95 or older	4.64 (1.78–12.13)	0.002			5.49 (2.09–14.39)	<0.001				
CFS (Not Frail)	Ref	-								
Frail	1.83 (1.36–2.45)	<0.001					2.12 (1.56–2.872.87)	<0.001		
mFI (Not-Frail)	Ref	-								
Frail	1.49 (1.13–1.96)	0.004							1.53 (1.16–2.03)	0.003
CFS (1–4)	Ref-	Ref-								
CFS 5	1.27 (0.84–1.90)	0.26								
CFS 6	1.88 (1.30–2.73)	0.001								
CFS 7–8	2.33 (1.62–3.45)	<0.001								

^&^ Base Model adjusted by sex, smoking status and elevated CRP.

**Table 3:** Longer Stay, mixed effects logistic regression, presenting the Odds ratio (OR) and adjusted OR (aOR) adjusted for the Clinical Frailty Scale (CFS), modified Frailty Index (mFI), and age group.

	Crude Odds Ratio (OR)		Base Model Adjusted OR (aOR) ^&^		Base + Age aOR		Base + CFS (Binary) aOR		Base + mFI (Binary) aOR	
	OR (95%CI)	*p*	aOR (95%CI)	*p*	aOR (95%CI)	*p*	aOR (95%CI)	*p*	aOR (95%CI)	*p*
Constant	2.64 (1.77–3.92)	<0.001	2.62 (1.62–4.23)	<0.001	1.74 (1.02–2.96)	0.04	1.47 (0.91–2.36)	0.12	2.22 (1.35–3.65)	0.002
Sex (Female)	Ref-									
Male	1.06 (0.79–1.41)	0.71	1.06 (0.79–1.42)	0.70	1.13 (0.84–1.73)	0.43	1.21 (0.90–1.64)	0.21	1.08 (0.81–1.45)	0.59
Smoking (Never/Ex)	Ref-									
Current smoker	0.69 (0.37–1.29)	0.25	0.69 (0.37–1.29)	0.25	0.75 (0.40–1.42)	0.38	0.60 (0.32–1.14)	0.12	0.69 (0.37–1.29)	0.25
Elevated CRP (≥40)	0.99 (0.73–1.35)	0.98	0.99 (0.73–1.35)	0.95	1.06 (0.78–1.46)	0.70	1.04 (0.76–1.43)	0.81	0.99 (0.72–1.35)	0.92
Age Group (65–74)										
75–84 yrs	1.23 (0.88–1.71)	0.23			1.24 (0.88–1.73)	0.22				
85–94 yrs	2.49 (1.64–3.79)	<0.001			2.52 (1.65–3.86)	<0.001				
95 or older	2.19 (0.70–6.84)	0.18			2.24 (0.71–7.04)	0.17				
CFS (Not Frail)	Ref-									
Frail	2.32 (1.71–3.14)	<0.001					2.44 (1.79–3.30)	<0.001		
mFI (Not-Frail)	Ref-									
Frail	1.39 (1.03–1.86)	0.03							1.39 (1.04–1.88)	0.028

^&^ Base Model adjusted by sex, smoking status and elevated CRP.

## Data Availability

The datasets used in the primary cohort analysed during the current study are available from the corresponding author on reasonable request on presentation of a statistical analysis plan addressing a new research question.

## Supplementary Materials

The following supporting information can be downloaded at: https://www.mdpi.com/article/10.3390/geriatrics7050087/s1, Figure S1: Scatterplot matrix of the Altman-Bland plots comparing the Clinical Frailty Scale (CFS), Age group and the modified Frail Index (mFI); Figure S2: Calibration plot of predicted and expected 28-day mortality in the external validation cohort using COPE models for (A) age, (B) CFS and (C) mFI; Figure S3: Calibration plots of predicted and expected prolonged admission in the external validation cohort using COPE models for (A) age, (B) CFS and (C) mFI; Table S1: A comparison between the Clinical Frailty Scale (CFS) and modified frailty index (mFI); Table S2: A comparison of Clinical Frailty Scale (CFS) and Age group; Table S3: Discrimination using an area under the receiver operating curve (AUROC) comparisons from the COPE data; Table S4: Baseline characteristics of the external validation cohorts; Table S5: Hosmer-Lemeshow goodness of fit measures for calibration in the external validation cohort. Note that a p-value < 0.05 and/or a high X-squared value indicates potential poor model calibration.
