# Supplementary material for: The Prognostic and Discriminatory Utility of the Clinical Frailty Scale and Modified Frailty Index Compared to Age
Source: Geriatrics (Basel). 2022 Aug 24;7(5):87. doi: 10.3390/geriatrics7050087 (PMC9498791; doi:10.3390/geriatrics7050087)

Supplementary Table S1: A comparison between the Clinical Frailty Scale (CFS) and modified frailty index (mFI)

		mFI						
**CFS**		0	0.2	0.4	0.6	0.8	1	**Total**
1-	Very Fit	11	6	3	0	0	0	**20**
2-	Fit	28	19	11	3	0	0	**61**
3-	Managing well	65	39	30	8	2	0	**144**
4-	Living with very mild frailty	47	38	46	12	0	0	**143**
5-	Living with mild frail	36	49	49	20	5	2	**161**
6-	Living with moderate frailty	40	56	70	40	9	3	**218**
7-	Living with severe frailty	27	37	89	33	8	2	**196**
8-	Living with very severe frailty	3	11	21	19	1	0	**55**
**Total**		**257**	**255**	**319**	**135**	**25**	**7**	**998**

Supplementary Table S2: A comparison of Clinical Frailty Scale (CFS) and Age group

		**Age Group (years)**				
**CFS**		65-74	75-84	85-94	≥95	**Total**
1-	Very Fit	15	3	2	0	**20**
2-	Fit	31	22	8	0	**61**
3-	Managing well	78	55	10	1	**144**
4-	Living with very mild frailty	51	69	21	2	**143**
5-	Living with mildly frail	27	79	51	4	**161**
6-	Living with moderate frailty	30	101	79	8	**218**
7-	Living with severe frailty	37	75	80	4	**196**
8-	Living with very severe frailty	18	19	16	2	**55**
**Total**		**287**	**423**	**267**	**21**	**998**

Supplementary Table S3: Discrimination using an area under the receiver operating curve (AUROC) comparisons from the COPE data

	**Age AUROC (95%CI)**	**p-value**	**CFS AUROC (95%CI)**	**p-value**	**mFI AUROC (95%CI)**	**p-value**
**28-Day mortality**						
Base Model[&]	0.65 (0.62-0.69)	<0.001	0.66 (0.62-0.69)	<0.001	0.62 (0.59-0.66)	0.21
Base Model[&] + Age			0.67 (0.64-0.71)	0.02	0.65 (0.62-0.69)	0.49
Base Model[&] + Age + mFI			0.67 (0.64-0.71)	0.03		

Base Model[&] + Age + CFS					0.67 (0.64-0.71)	0.83
Base Model[&] + CFS	0.67 (0.64-0.71)	0.055			0.66 (0.62-0.69)	0.94
**Prolonged admission**						
Base Model[&]	0.60 (0.56-0.63)	0.007	0.66 (0.62-0.70)	<0.001	0.56 (0.52-0.60)	0.053
Base Model[&] + Age			0.68 (0.64-0.71)	<0.001	0.61 (0.58-0.65)	0.13
Base Model[&] + Age + mFI			0.68 (0.64-0.71)	<0.001		
Base Model[&] + Age + CFS						
Base Model[&] + CFS	0.68(0.64-0.71)	0.06			0.68 (0.64-0.71)	0.07

[&]Note: Base model comprised of Sex, Smoking status and CRP (≥40)

Supplementary Table S4: Baseline characteristics of the external validation cohorts.

	**Edinburgh**			**Cambridge***		
	**All n = 461**	**Alive at 28 days n = 285**	**Dead at 28 days n = 176**	**All n=213**	**Alive at 28 days n=140**	**Dead at 28 days n=73**
**Age Group (years)**						
65-74	135 (29)	93 (33)	42 (24)	52 (24)	43 (31)	9 (12)
75-79	85 (18)	59 (21)	26 (15)	53 (25)	37 (26)	16 (22)
80-84	97 (21)	55 (19)	42 (24)	39 (18)	23 (16)	16 (22)
85-89	78 (17)	46 (16)	32 (18)	35 (16)	17 (12)	18 (25)
90 or older	66 (14)	32 (11)	34 (19)	34 (16)	20 (14)	14 (19)
Female sex	209 (45)	141 (49)	68 (39)	94 (44)	68 (49)	26 (36)
**Smoking status**						
Never/ex-smoker	439 (95)	269 (94)	170 (97)	173 (81)	116 (83)	57 (78)
Current smoker	22 (5)	16 (6)	6 (3)	14 (7)	9 (6)	5 (7)
Missing	–	–	–	26 (12)	15 (11)	11 (15)
**Comorbidities**						
Diabetes	113 (25)	68 (24)	45 (26)	58 (27)	43 (31)	15 (21)
Hypertension	229 (50)	140 (49)	89 (51)	101 (47)	70 (50)	31 (43)
Coronary artery disease	–	–	–	57 (27)	36 (26)	21 (29)
Myocardial infarction	53 (11)	30 (11)	23 (13)	–	–	–
Heart failure	66 (14)	37 (13)	29 (16)	40 (19)	19 (14)	21 (29)
COPD	87 (19)	55 (19)	32 (18)	41 (19)	26 (19)	15 (21)
**Maximal CRP>40**	354 (77)	210 (74)	144 (82)	146 (69)	88 (63)	58 (80)
**MFI ≥ 0.4**	221 (48)	128 (45)	93 (53)	117 (55)	74 (53)	43 (59)
**CFS Score**						
1-4	185 (40)	131 (46)	54 (31)	72 (34)	57 (41)	15 (21)
5	82 (18)	49 (17)	33 (19)	32 (15)	22 (16)	10 (14)
6	133 (29)	78 (27)	55 (31)	54 (25)	31 (22)	23 (32)
7-8	61 (13)	27 (10)	34 (19)	55 (26)	30 (21)	25 (34)

Values are n (%). *2 patients from the Cambridge cohort were excluded from the validation analysis due to missing CRP values.

Abbreviations: COPD = chronic obstructive pulmonary disease; CRP = C-reactive protein; mFI = modified frailty index; CFS = clinical frailty scale.

Supplementary Table S5: Hosmer-Lemeshow goodness of fit measures for calibration in the external validation cohort. Note that a p-value <0.05 and/or a high X-squared value indicates potential poor model calibration.

**COPE Model**	**HL-Test p-value**	**HL-Test X-squared**
28-day mortality		
Age	0.36	8.7
CFS	0.12	12.7
mFI	0.34	9.1
Prolonged admission		
Age	0.30	9.6
CFS	0.62	6.2
mFI	0.24	10.3

Supplementary Figure S1.

Scatterplot matrix of the Altman-Bland plots comparing the Clinical Frailty Scale (CFS), Age group and the modified Frail Index (mFI)

	**CFS**	**Age Group**
**Age Group**	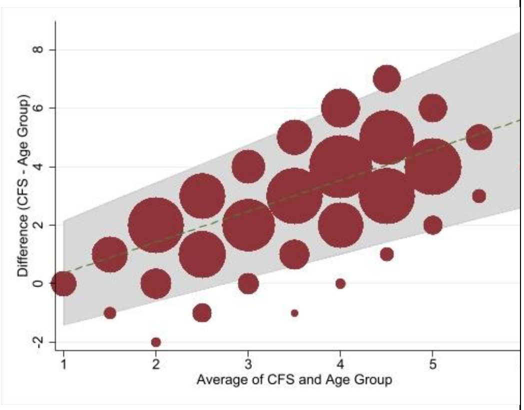	
**mFI**	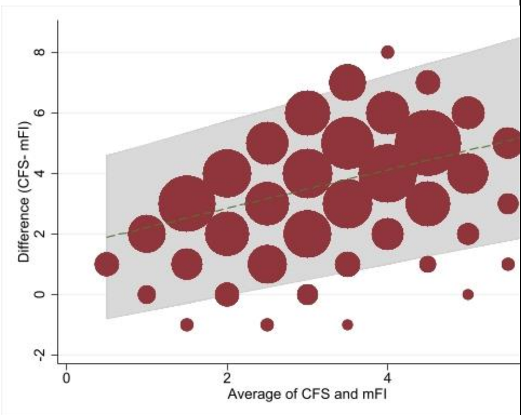	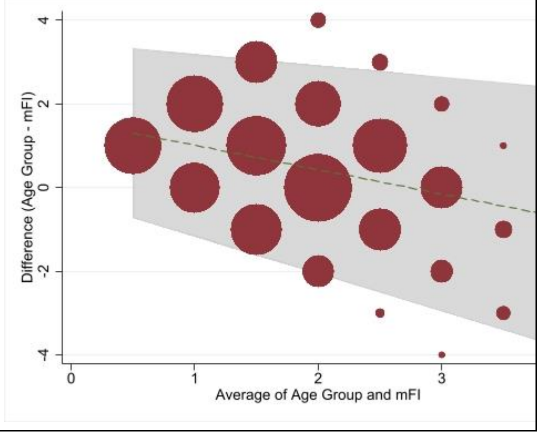

Supplementary Figure S2. Calibration plot of predicted and expected 28-day mortality in the external validation cohort using COPE models for (A) age, (B) CFS and (C) mFI.

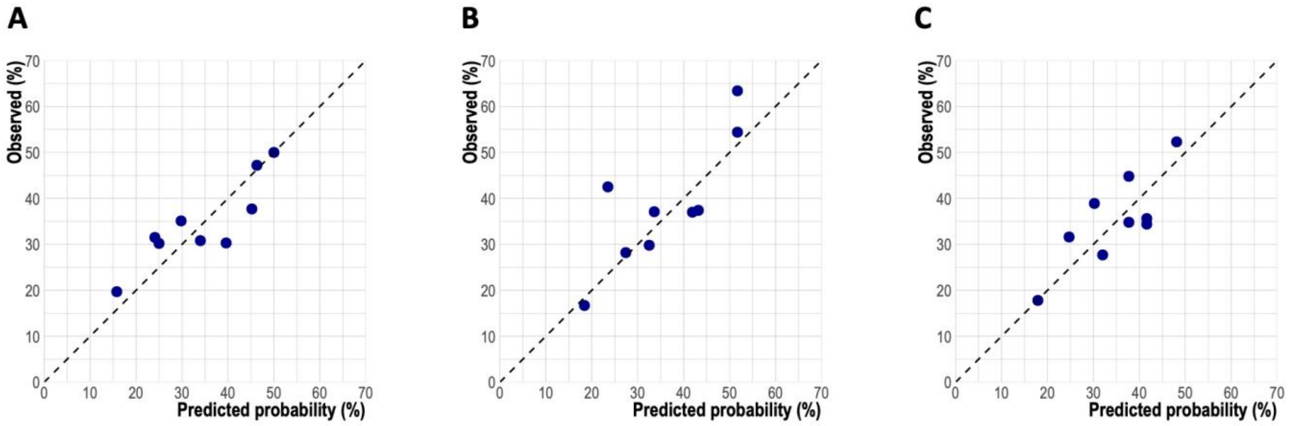

Supplementary Figure S3. Calibration plots of predicted and expected prolonged admission in the external validation cohort using COPE models for (A) age, (B) CFS and (C) mFI.

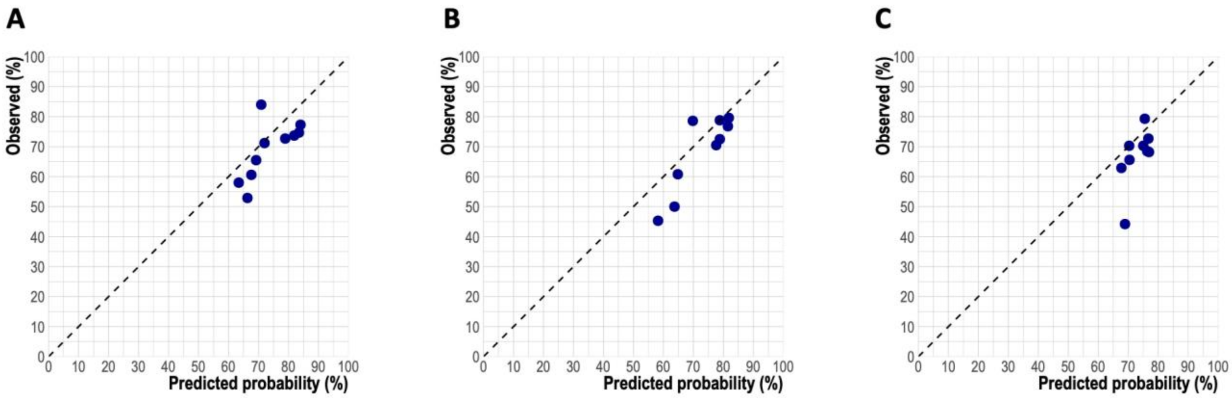

Supplement: Supplementary file 1 [file geriatrics-07-00087-s001.zip › geriatrics-1839662-supplementary.pdf]
